# Quantifying geocode location error using GIS methods

**DOI:** 10.1186/1476-069X-6-10

**Published:** 2007-04-04

**Authors:** Matthew J Strickland, Csaba Siffel, Bennett R Gardner, Alissa K Berzen, Adolfo Correa

**Affiliations:** 1 National Center on Birth Defects and Developmental Disabilities, Centers for Disease Control and Prevention, Atlanta, Georgia, USA; 2 Battelle Centers for Public Health Research and Evaluation, Atlanta, Georgia, USA; 3 Computer Sciences Corporation, Atlanta, Georgia, USA; 4 Agency for Toxic Substances and Disease Registry, Centers for Disease Control and Prevention, Atlanta, Georgia, USA

## Abstract

**Background:**

The Metropolitan Atlanta Congenital Defects Program (MACDP) collects maternal address information at the time of delivery for infants and fetuses with birth defects. These addresses have been geocoded by two independent agencies: (1) the Georgia Division of Public Health Office of Health Information and Policy (OHIP) and (2) a commercial vendor. Geographic information system (GIS) methods were used to quantify uncertainty in the two sets of geocodes using orthoimagery and tax parcel datasets.

**Methods:**

We sampled 599 infants and fetuses with birth defects delivered during 1994–2002 with maternal residence in either Fulton or Gwinnett County. Tax parcel datasets were obtained from the tax assessor's offices of Fulton and Gwinnett County. High-resolution orthoimagery for these counties was acquired from the U.S. Geological Survey. For each of the 599 addresses we attempted to locate the tax parcel corresponding to the maternal address. If the tax parcel was identified the distance and the angle between the geocode and the residence were calculated. We used simulated data to characterize the impact of geocode location error. In each county 5,000 geocodes were generated and assigned their corresponding Census 2000 tract. Each geocode was then displaced at a random angle by a random distance drawn from the distribution of observed geocode location errors. The census tract of the displaced geocode was determined. We repeated this process 5,000 times and report the percentage of geocodes that resolved into incorrect census tracts.

**Results:**

Median location error was less than 100 meters for both OHIP and commercial vendor geocodes; the distribution of angles appeared uniform. Median location error was approximately 35% larger in Gwinnett (a suburban county) relative to Fulton (a county with urban and suburban areas). Location error occasionally caused the simulated geocodes to be displaced into incorrect census tracts; the median percentage of geocodes resolving into incorrect census tracts ranged between 4.5% and 5.3%, depending upon the county and geocoding agency.

**Conclusion:**

Geocode location uncertainty can be estimated using tax parcel databases in a GIS. This approach is a viable alternative to global positioning system field validation of geocodes.

## Background

Federal, state, and local public health surveillance systems often collect residential address information as part of their surveillance activities. Prior to spatial statistical analyses, residential address information must be geocoded (e.g., latitude and longitude coordinates), a process typically accomplished through the use of electronic street databases [1,2]. Public health applications of geocoded data include defining a study population, linking health outcomes with environmental hazards, and investigating disease clusters. Although the hope is that all geocodes correctly reflect the true geographic location of the addresses, some geocodes are likely inaccurate due to errors in street databases, errors in residential address information, algorithms that permit imperfect address matches (i.e., the "match rate," or how similar the submitted address must be to the address in the database), and the distance geocodes are placed from the street centerline [2-5]. There is generally a trade-off between the proportion of missing geocodes and geocode accuracy; lenient match rates tend to increase the proportion of successfully geocoded addresses at the expense of geocode accuracy [6]. In addition to carefully collected residential address information, street databases that are current, free of errors, and spatially accurate should help reduce location error [2].

Because geocode inaccuracies can affect spatial analyses, [7] understanding the magnitude of location error in geocoded data is desirable. One approach is to travel to the address location and verify coordinates using a global positioning system (GPS) [1,2,8-10]. Although this approach is accurate it is also resource-intensive, particularly when the geographic area of interest is large. Whereas a GPS may be the only viable option for geocoding in remote settings [e.g., [11]], in the U.S. alternative options for geocode validation are generally available, and those overseeing surveillance systems may not wish to, or have the resources to, verify large numbers of addresses using a GPS. In this paper we describe an alternative computer-based method [5] to verify address locations for a sample of birth defect records in metropolitan Atlanta.

## Methods

### Population and sample

The Metropolitan Atlanta Congenital Defects Program (MACDP) is a population-based birth defects surveillance system operated by Centers for Disease Control and Prevention since 1968 [12]. MACDP actively ascertains infants and fetuses with birth defects born to mothers residing in one of five metropolitan Atlanta counties at delivery (Clayton, Cobb, DeKalb, Fulton, and Gwinnett). The address of the maternal residence is recorded for each case and is subsequently sent to a commercial vendor for geocoding. Independent of MACDP geocoding efforts, the Office of Health Information and Policy (OHIP), Georgia Division of Public Health, has geocoded the live birth cohort in Georgia since 1994.

The initial phase of geocoding is similar for both the commercial vendor and OHIP. Although the tolerance for accepting imperfect matches may differ, both agencies begin by comparing (in batch mode) submitted addresses with addresses in a street database. The commercial vendor uses street databases distributed by Geographic Data Technology (now Tele Atlas), whereas OHIP uses street databases distributed by Group 1 software. Street databases contain many road segments; each segment has two address ranges (one side of the road has an even numbered address range and the other side has an odd numbered address range). If the submitted address falls within a range then a geocode is generated by interpolating between the two known addresses at opposite ends of the road segment. When a street-level match cannot be achieved the software assigns a geocode corresponding to a polygon centroid. The commercial vendor accepts centroid matches up to the 5-digit ZIP code level and OHIP accepts centroid matches up to the census tract level. After batch geocoding, the commercial vendor manually compares each address not successfully geocoded to a list of potential addresses. If a potential address is judged to be a reasonable match the record is manually geocoded. OHIP performs a spatial imputation on addresses that are not geocoded successfully [13]. Imputation begins by estimating the county for all remaining addresses. Using vital records data, the expected number of births by race and census tract is calculated for each county. Addresses are imputed into census tracts that have less than the expected number of geocoded records and are assigned the corresponding centroid.

As part of ongoing surveillance activities, MACDP links its birth defects records with OHIP records using a deterministic approach based on several variables including names, dates, and addresses. As a result, each successfully linked record has two independently created geocodes – one OHIP geocode and one commercial vendor geocode. We defined the study population, based on MACDP records, as all infants with birth defects delivered during 1994–2002 with maternal residence at delivery in Fulton or Gwinnett County. From this study population, we randomly selected 665 records meeting the following criteria: 1) successful link with OHIP records, 2) address on the MACDP record matched address on the OHIP record, and 3) both OHIP and the commercial vendor attempted to geocode the address. This study was approved by the CDC institutional review board and was conducted in accordance with the Declaration of Helsinki of the World Medical Association.

### Geographic data

A "shapefile" is a set of computer files used to store geographic information (e.g., census tract boundaries) and tables of attributes associated with the geographic information (e.g., census tract housing and demographic characteristics) [14]. Shapefiles can be manipulated using a geographic information system (GIS); ArcView 8.3 (ESRI, Redlands, CA) was used in this project. Tax parcel shapefiles were obtained from the Fulton and Gwinnett County tax assessor's offices. These shapefiles contain polygons corresponding to the location and dimensions of each taxable land parcel in the county. The address of each parcel is stored in its attribute table.

We also obtained high-resolution (0.3 meter resolution per pixel) digital orthoimages from the U.S. Geological Survey (USGS). An orthoimage is a remotely sensed digital photograph of the earth's surface that has been mathematically manipulated to minimize distortion due to terrain relief and sensor orientation [15]. USGS estimates that the design accuracy of its orthoimages does not exceed a root mean squared error of 3-meters in diagonal [15].

### Location error assessment

For each of the 665 records we attempted to identify the tax parcel corresponding to the maternal address. When the parcel was identified, a point was placed on the residence located within the parcel. We elected not to place points when tax parcels contained many buildings (i.e., large apartment complexes) because there was no obvious location for point placement. During validation, we identified a subset of records that presumably had the incorrect county recorded in the MACDP database. We examined these addresses further using the U.S. Postal Service online lookup database to infer the correct county. After excluding records with incorrect county codes (n = 66), the final sample consisted of 599 records.

**Figure 1:**
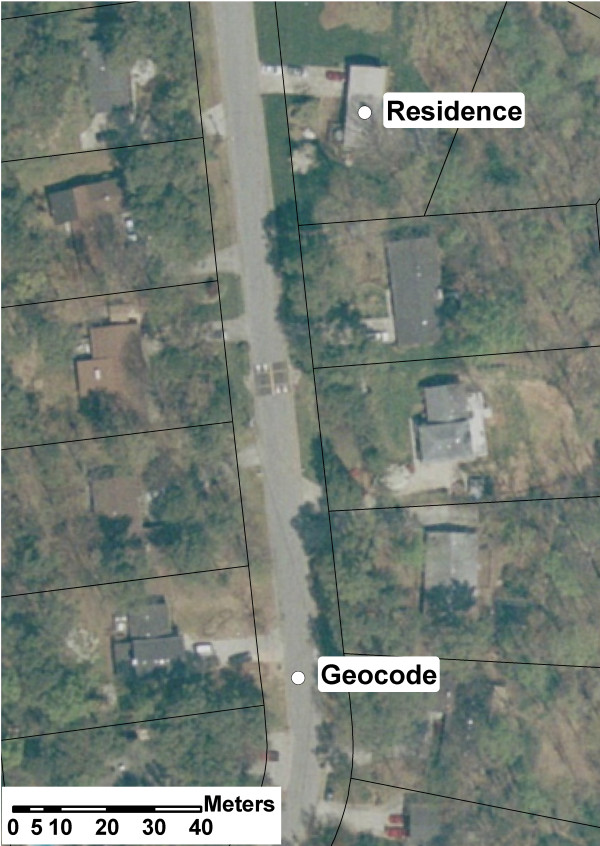
**Tax Parcel Data Overlain on Orthoimagery**. The distance between the geocode and the residence (gold standard) is the "location error" for the address.

The geographic coordinates of the placed points were determined using ArcView and represent the "gold standard." For each validated address, we calculated both the distance and the angle between the gold standard and each of the two geocodes applying a spherical earth model [16]. We assumed a constant elevation of 300 meters above sea level. Figure 1 displays tax parcel data overlain on orthoimagery; we define location error as the distance between the geocode and the gold standard (residence). We report the empirical cumulative distribution of location errors for both the OHIP and commercial vendor geocodes. Rose plots were generated to inspect whether the distribution of angles appeared uniform, and Rayleigh tests were performed to evaluate the null hypothesis of a uniform circular distribution of angles. We created rose plots by stratifying addresses according to the angle of the location error. Each stratum, or "bin," correspond to a 15° increment (i.e., 0°-15°, 15°-30°, etc.). Each bin has its own "petal," which varies in size according to the number of addresses within the bin.

### Census tract point-in-polygon simulations

To characterize the impact of geocode location error on census tract assignment we generated 5,000 random geocodes within each county and used a point-in-polygon routine to determine the Census 2000 tract for each geocode. Each geocode was then displaced at a random angle from a uniform (0, 2π) distribution by a random distance drawn from an empirical distribution of geocode location errors (as reported in the Results). We then determined the census tract for each displaced geocode. We conducted 5,000 such simulations for each geocoding agency within each county and we report the percentage of geocodes that resolved into the incorrect census tract (median, 2.5%, and 97.5% of the 5,000 simulations). All simulations were performed using the Universal Transverse Mercator (UTM) Zone 16 North map projection with the software package R 2.4.0 (R Core Development Team).

## Results

The commercial vendor and OHIP created address-level geocodes for 96.0% and 91.7% of the sample, respectively (Table 1). Although 435 addresses included in the sample were located (72.6%), gold standard points were placed for only 376 addresses (62.8%). Points were not placed for 59 addresses (9.8%) because the parcels contained large, multi-unit housing complexes.

**Table 1:** Frequencies of Geocoding Success and Geocode Validation Outcomes for 599 Selected Addresses, by County.

	Fulton County (n = 339)		Gwinnett County (n = 260)		Both Counties (n = 599)	
	
	n	%	n	%	n	%
Address-level geocodes						
OHIP	321	94.7	228	87.7	549	91.7
Commercial vendor	324	95.6	251	96.5	575	96.0
Addresses located using GIS						
House/small multi-unit complex	194	57.2	182	70.0	376	62.8
Moderate/large multi-unit complex	41	12.1	18	6.9	59	9.8
Unverified addresses	104	30.7	60	23.1	164	27.4

**Figure 2:**
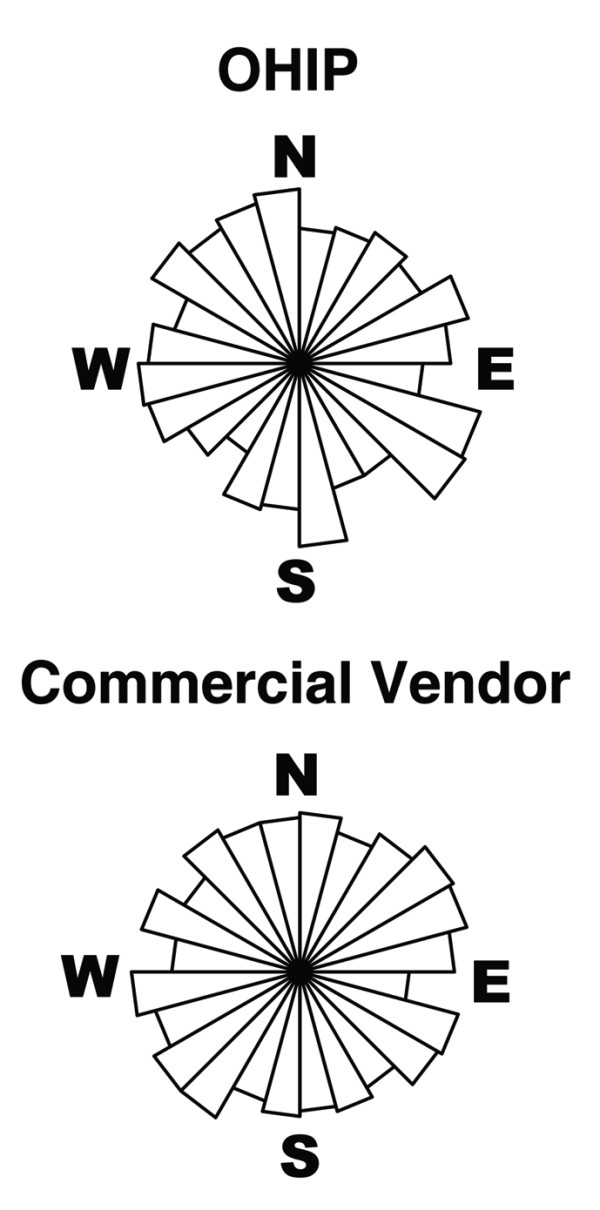
**Distributions of Geocode Location Error Angles**. Rose plots portraying the distribution of angles between the geocode and the residence (using 15° bins) for OHIP and commercial vendor geocodes.

Selected percentiles from the empirical cumulative distributions of location error, stratified by county, are presented for both OHIP and commercial vendor geocodes in Table 2. Median location error was 71 meters for the commercial vendor and 91 meters for OHIP geocodes. Median location error was approximately 35% greater in Gwinnett County than in Fulton County. This finding was anticipated, as Gwinnett County is predominantly suburban whereas Fulton County has a mix of urban and suburban areas. Rose plots (Figure 2) were constructed by placing each record into one of 24 15° bins according to the angle of the location error. Inspection of the rose plots did not suggest systematic bias in the direction of the geocode relative to the gold standard. There was no strong evidence to reject the null hypothesis of a uniform circular distribution. Rayleigh tests, which were performed for each geocoding agency (data pooled over counties) as well as for each combination of county and geocoding agency, were not significant (all p-values > 0.2).

**Table 2:** Selected Percentiles From the Empirical Cumulative Distributions of Location Error. All distances reported in meters.

	Fulton County		Gwinnett County		Both Counties	
Percentile	Commercial vendor (n = 189)	OHIP (n = 189)	Commercial vendor (n = 178)	OHIP (n = 169)	Commercial vendor (n = 367)	OHIP (n = 358)

25%	39	38	44	61	42	48
50%	61	77	84	104	71	91
75%	124	136	141	171	147	155
90%	242	281	311	311	281	301
95%	322	361	378	373	352	369
99%	573	693	747	738	664	774
Max	1,389	20,677	1,500	2,324	1,500	20,677

The magnitude of location error reported in Table 2 occasionally causes geocodes to be placed into incorrect census tracts. The point-in-polygon simulations for Fulton County using the commercial vendor location error caused 4.5% (4.0%, 5.0%) of the randomly generated geocodes to be placed into incorrect census tracts. OHIP location error caused incorrect census tract assignment for 5.3% (4.8%, 5.9%) of the geocodes in Fulton County. Results were similar for Gwinnett County; 4.8% (4.3%, 5.4%) of geocodes were placed into incorrect census tracts because of commercial vendor location error, and OHIP location error caused 5.2% (4.7%, 5.8%) of geocodes to be assigned the incorrect census tract.

## Discussion

Location error is intrinsic to both the OHIP and commercial vendor geocodes. Interpolation is likely a major component of this error, as interpolation is necessary whenever the submitted address is not demarcated on the street map. Because only a small proportion of addresses are demarcated, interpolation occurs frequently. Observed differences in geocoding success and in location error magnitude between the commercial vendor and OHIP geocodes may be due to a number of factors, including the quality of the street database, the correctness of the submitted addresses, the ability of the software to match submitted addresses with addresses in the database (e.g., recognize that "Cir" is short for "Circle"), the tolerance for geocoding imperfect matches (i.e., the match rate), and the methodology used to geocode addresses that were not geocoded in batch mode. Although we were unable to quantify the relative contribution of each of these factors, it is likely that much of the difference in the percentage of addresses successfully geocoded is attributable to the manual address matching performed by the commercial vendor.

Although the aim of our study was to estimate the distributions of geocode location error, there are additional errors to consider when analyzing geocoded address data. The addresses unsuccessfully geocoded by the commercial vendor and/or OHIP (and therefore excluded from analyses) may result in selection bias. If the probability that a geocode is missing is differential across space then this can bias the relationship between spatially-varying covariates and disease incidence [17,18]. Furthermore, a high proportion of successfully geocoded addresses, although reassuring, does not preclude this selection bias. An additional source of error arises when spurious geocodes are created from fictitious addresses. This can occur when an imperfectly recorded address happens to fall within a range of viable addresses.

Our study design excluded discordant addresses in the linked MACDP and OHIP database. This approach ensured that comparisons between the two sets of geocodes were fair and presumably reduced the number of low quality addresses that were validated (an address that is identical in both the MACDP and OHIP databases is likely to be correct). This design, however, may have underestimated the true distribution of geocode location error for both commercial vendor and OHIP geocodes. Many imperfect addresses, which were excluded from our study (because the MACDP and OHIP addresses were discordant), were nevertheless geocoded to the address-level by both agencies. The distribution of geocode location error for these addresses may be large relative to the distribution for the set of addresses selected for validation. Additionally, the 164 addresses selected for validation that we were unable to locate in the tax parcels (Table 1) frequently had address-level geocodes. Although it is probable that many of these addresses correspond to apartment complex roads not delineated in the shapefile, the true distribution of geocode location error for these geocodes may be larger than the distributions presented in Table 2.

We used a simulation-based approach to evaluate the potential impact of location error on census tract assignment. The percentage of geocodes displaced into incorrect census tracts was similar for Fulton County and Gwinnett County, even though median location error was approximately 35% larger in Gwinnett County (Table 2). This finding is likely due to census tract size (tracts tend to be larger in the predominantly suburban Gwinnett County). A larger location error is needed to displace a geocode outside of its original census tract in Gwinnett.

Presumably, the ramifications of geocode location error will vary depending upon the study design. For example, in air pollution epidemiology, designs using ambient air quality monitors to assign pollution levels to cohort members [e.g., [19]] may not be strongly impacted by the magnitude of geocode location error reported in Table 2, whereas fine-scale studies of traffic proximity [e.g., [20]] may be strongly impacted by this magnitude of location error. In both of these settings, however, an empirical estimate of geocode location error, specific to the local setting in which the study was conducted, can be used to formally evaluate the consequences of this source of measurement error on epidemiologic results. Tenuous speculations about measurement error can be replaced with inferences from more rigorous statistical approaches.

Relative to street databases, tax parcel shapefiles offer two main advantages: 1) fictitious addresses that happen to fall within ranges of legitimate addresses are not geocoded, and 2) there is no need to interpolate between address ranges. Tax parcels, however, have certain disadvantages as well. They are created to assist the county tax assessor rather than to geocode addresses. Accordingly, an apartment complex encompassing numerous roads appears as one polygon because this is the taxable parcel. We were unable to locate 164 addresses (Table 1), and it is probable that many of these addresses correspond to apartment complex roads not delineated in the shapefile. An additional disadvantage is the limited availability of tax parcel shapefiles (as of June 2005 only two of the five counties covered by MACDP had tax parcel shapefiles). Some GIS software packages offer capabilities for batch parcel geocoding (e.g., the "One Field" style of locator in ArcGIS); as tax parcel shapefiles become increasingly available parcel-based geocoding may become more feasible. Building "footprints," where polygons in the shapefile correspond to building dimensions, also offer possibilities for geocoding and geocode validation.

Cost is also an important consideration – whereas 25–30 addresses per hour can be validated using tax parcels, online commercial geocoding services offer near real-time geocoding for less than two cents per address. Tax parcels are therefore not a viable alternative to batch street database geocoding. Tax parcel validation, however, is more efficient than GPS field validation. Although the number of addresses per hour that can be field validated will vary greatly depending upon address proximity, it would be nearly impossible to field validate 25 addresses per hour in Atlanta. Past experiences at MACDP suggest rates of 5–10 addresses per hour are more typical.

Applications of tax parcel datasets in environmental health extend beyond geocode generation and validation. For example, tax parcel datasets and housing characteristics have been combined to identify high priority regions of lead poisoning risk [21]. The 2004 Olympic and Para Olympic environmental health inspection program [22] utilized tax parcel data in their GIS applications. Tax parcel datasets are also routinely used in urban planning, and some investigators have used tax parcels to model the environmental impact of urban development [23].

## Conclusion

Geocode uncertainty can be quantified using tax parcel datasets, high resolution orthoimagery, and GIS. In metropolitan Atlanta, the median geocode location error was less than 100 meters for both the OHIP and commercial vendor geocodes, and there was no evidence of systematic bias in the angle of the location error. Geocode location error caused approximately 5% of the randomly generated geocodes to be placed into the incorrect census tract. We contend that the motivation for understanding the distribution of geocode location error parallels the motivation for assessing disease misclassification or exposure measurement error in epidemiological studies. Geocodes have an important role in environmental health research and surveillance, as they are frequently used to define the study population and to link health data with environmental hazards. Furthermore, many spatial statistical methods use geocodes, and the validity of these approaches may be compromised by location error. Further work is needed to evaluate the impact of location error on statistical methods and surveillance applications.

## Competing interests

The author(s) declare that they have no competing interests.

## Authors' contributions

MJS conducted the addresses validation and drafted the manuscript. CS helped design the study, draft the manuscript, and is responsible for the maintenance of MACDP geocoded data. BRG examined a subset of records to verify whether the addresses fell within the counties and assisted with the creation of Figures. AKB was responsible for obtaining the tax parcel datasets, working with the dataset owners to ensure appropriate use of the datasets, and assisting MJS and CS with GIS activities. AC helped design the study and participated in its coordination. All authors read and approved the final manuscript.
